# A Novel Combination Cancer Therapy with Iron Chelator Targeting Cancer Stem Cells via Suppressing Stemness

**DOI:** 10.3390/cancers11020177

**Published:** 2019-02-03

**Authors:** Yuki Katsura, Toshiaki Ohara, Kazuhiro Noma, Takayuki Ninomiya, Hajime Kashima, Takuya Kato, Hiroaki Sato, Satoshi Komoto, Toru Narusaka, Yasuko Tomono, Boyi Xing, Yuehua Chen, Hiroshi Tazawa, Shunsuke Kagawa, Yasuhiro Shirakawa, Tomonari Kasai, Masaharu Seno, Akihiro Matsukawa, Toshiyoshi Fujiwara

**Affiliations:** 1 Department of Gastroenterological Surgery, Okayama University Graduate School of Medicine, Dentistry and Pharmaceutical Sciences, Okayama 700-8558, Japan kyuki82@yahoo.co.jp (Y.K.); knoma@md.okayama-u.ac.jp (K.N.); nino_chan@msn.com (T.N.); hkashima@s.okayama-u.ac.jp (H.K.); alternative_winter_0203@yahoo.co.jp (T.K.); hiromaiaoi@gmail.com (H.S.); CometHeart13@gmail.com (S.K.); narusakat@gmail.com (T.N.); htazawa@md.okayama-u.ac.jp (H.T.); skagawa@md.okayama-u.ac.jp (S.K.); yasuwr@md.okayama-u.ac.jp (Y.S.); toshi_f@md.okayama-u.ac.jp (T.F.); 2 Department of Pathology and Experimental Medicine, Okayama University Graduate School of Medicine, Dentistry and Pharmaceutical Sciences, 2-5-1 Shikata-cho, Kita-ku, Okayama 700-8558, Japan; xingboyi1991@gmail.com (B.X.); CynthiaChen9408@gmail.com (Y.C.); amatsu@md.okayama-u.ac.jp (A.M.); 3 Shigei Medical Research Institute, Okayama 701-0202, Japan; yatomono@mx3.tiki.ne.jp; 4 Center for Innovative Clinical Medicine, Okayama University Hospital, Okayama 700-8558, Japan; 5 School of Bioscience and Biotechnology, Tokyo University of Technology, Tokyo 192-0914, Japan; kasaitnr@stf.teu.ac.jp; 6 Laboratory of Nano-Biotechnology, Okayama University Graduate School of Interdisciplinary Science and Engineering in Health Systems, Okayama 700-8530, Japan; mseno@okayama-u.ac.jp

**Keywords:** cancer stem cells, stemness, iron, combination therapy

## Abstract

Excess iron causes cancer and is thought to be related to carcinogenesis and cancer progression including stemness, but the details remain unclear. Here, we hypothesized that stemness in cancer is related to iron metabolism and that regulating iron metabolism in cancer stem cells (CSCs) may be a novel therapy. In this study, we used murine induced pluripotent stem cells that expressed specific stem cell genes such as *Nanog*, *Oct3/4*, *Sox2*, *Klf4*, and *c-Myc*, and two human cancer cell lines with similar stem cell gene expression. Deferasirox, an orally available iron chelator, suppressed expression of stemness markers and spherogenesis of cells with high stemness status in vitro. Combination therapy had a marked antitumor effect compared with deferasirox or cisplatin alone. Iron metabolism appears important for maintenance of stemness in CSCs. An iron chelator combined with chemotherapy may be a novel approach via suppressing stemness for CSC targeted therapy.

## 1. Introduction

Iron is an essential element and plays crucial roles in our body, including roles in cell growth, proliferation, DNA synthesis, and energy metabolism. On the other hand, excess iron is associated with tumorigenesis in many types of human cancers [1,2,3] and is also associated with cancer progression. These indicate that iron is an essential element for cancer cells and thought to be a therapeutic target. Thus, iron depletion through chelation and an iron-deficient diet have been explored as possible therapeutic interventions in various types of cancer [4,5,6]. Our group has also shown the antitumor effect of iron depletion therapy using an iron-deficient diet [7]. 

According to the cancer stem cell (CSC) hypothesis, CSCs exist in many types of cancer tissues and are considered resistant to conventional types of therapy such as chemotherapy and radiation therapy. CSCs are also related to recurrence and metastasis. CSCs have been reported in various types of cancer [8,9,10,11,12]. Therapy targeting CSCs has been explored recently, but effective CSC therapy has not been established. Iron is known to be essential for cancer and associated with tumor tumorigenesis and cancer progression, which suggest us the existence of relationship between iron metabolism and CSCs. Thus, we hypothesized that cancer stemness, which is strongly related to cancer malignancy, may also be strongly related to iron metabolism and that iron depletion therapy may be a novel approach to target CSCs.

CSCs possess the features of normal stem cells, including self-renewal and pluripotency, in addition to cancer cell features. CSCs are distinguished by the expression of stemness markers. From the viewpoint of stem cell hierarchy, embryonic stem cells (ES cells) possess the properties of pluripotency and self-renewal and are at the top of the stem cell hierarchy. Several transcription factors, including Nanog, Oct3/4, Sox2, Klf4, and c-Myc, regulate the stemness of ES cells and are also upregulated in various types of CSCs [13,14,15,16,17]. Therefore, our group selected murine induced pluripotent stem cells (miPS cells), which possess similar properties as ES cells, as a model of cells with high stemness status and verified the effect of iron chelation using deferasirox (DFX) against stemness. Furthermore, our group selected human cancer cell lines that express the same stemness markers as ES cells as a model of CSCs. We verified the effect against stemness and evaluated the effectiveness of combination therapy with DFX plus chemotherapy using cisplatin (CDDP).

## 2. Results

### 2.1. DFX Suppresses Expression of Stemness Markers and Spherogenicity of miPS Cells

**Figure 1:**
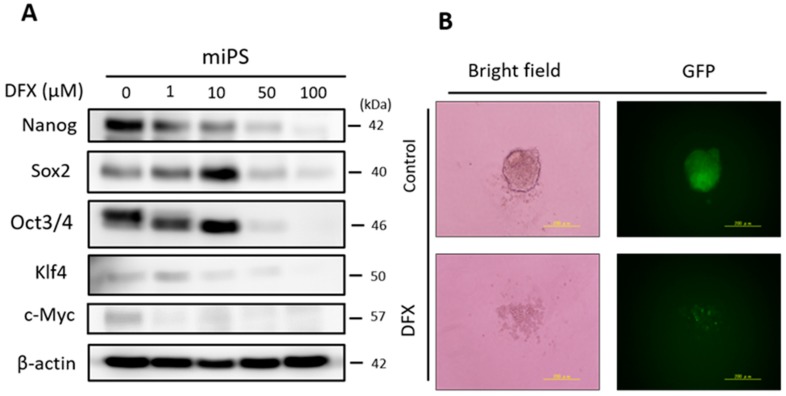

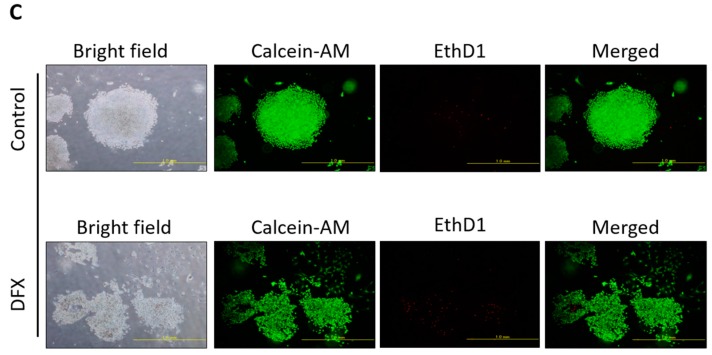
The effect of DFX against stemness of miPS cells in vitro and cytotoxicity analysis. (**A**) miPS cells were treated with the indicated dose of DFX (0, 1, 10, 50, 100 μM) and subjected to western blot analysis with antibodies to stemness markers (Nanog, Sox2, Oct3/4, Klf4, c-Myc) or β-actin (loading control). Stemness markers were suppressed by DFX at concentrations over 50 μM. (**B**) miPS cells treated with 50 μM DFX were cultured in suspension for 72 h. DFX treatment of miPS cells suppressed spherogenesis and GFP expression, which indicates suppression of Nanog. (**C**) Micrographs of the fluorescence-based Live/Dead assay showing live and dead miPS cells following treatment with 0.2% DMSO (control) or 50 μM DFX (magnification ×40). The morphology of miPS cells after treatment with DFX changed from round to spindle shaped. Almost all cells were stained green, which indicates live cells.

To evaluate the effect of DFX on expression of stemness markers in miPS cells, the cells were cultured with several concentrations of DFX (0, 1, 10, 50, 100 μM). DFX suppressed expression of stemness markers at concentrations over 50 μM (Figure 1A). To evaluate the effect of DFX on spherogenicity, a sphere formation assay was performed. DFX suppressed the spherogenicity of miPS cells (Figure 1B). To assess the effect of DFX on cytotoxicity and morphologic changes, the Live/Dead assay was performed. The morphology of some miPS cells changed to spindle shaped, but almost all cells were alive after DFX treatment (Figure 1C). These results indicate that DFX suppresses the stemness properties of miPS cells but does not induce substantial cytotoxicity.

### 2.2. DFX Suppresses Tumorigenicity and Expression of Tumor Stemness Markers in miPS Cells In Vivo

To address the effect of DFX on tumorigenicity and expression of stemness markers in vivo, we employed a subcutaneous allograft model by using BALB/c nude mice. miPS cells were treated with 0.2% dimethyl sulfoxide (DMSO) for 48 h as the control group or with 50 μM DFX for 48 h as the DFX group and then injected into the right flank. 

**Figure 2:**
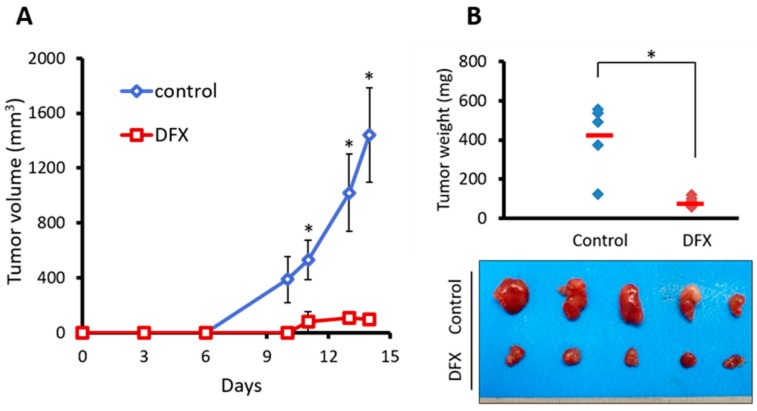

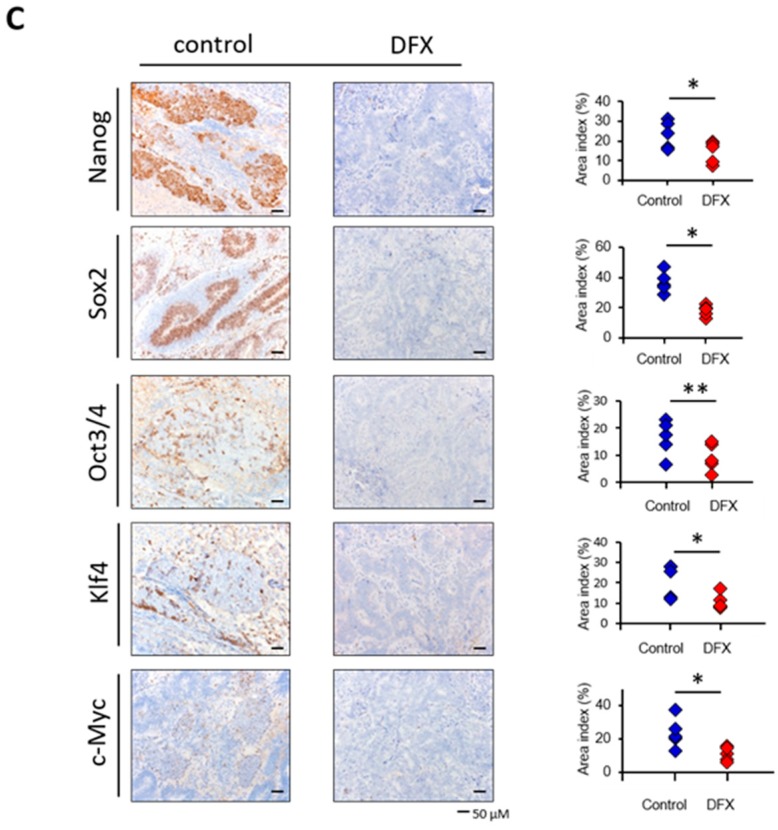
DFX suppressed tumorigenicity and expression of stemness markers in miPS cells in vivo. (**A**) miPS cells (5 × 10^5^ per mouse) treated with 0.2% DMSO or 50 μM DFX were implanted subcutaneously into the right flank, and tumorigenicity was evaluated. DFX significantly suppressed the tumor volume of miPS cells in vivo. * *p* < 0.05. (**B**) DFX significantly suppressed the tumor weight of miPS cells in vivo. * *p* < 0.05. Macroscopic images show that tumors in the DFX group were smaller than those in the control group. (**C**) Harvested tumors were analyzed for expression of stemness markers (Nanog, Sox2, Oct/4, Klf4, c-Myc) by immunohistochemistry, and evaluation of the stemness marker area index was calculated with Image J software. * *p* < 0.05, ** *p* = 0.09. Most stemness markers, except Oct3/4, were significantly suppressed in the DFX group.

Tumorigenesis was observed. Fourteen days after injection, tumors were harvested, and the tumor volume and immunohistochemistry of stemness markers were evaluated. The tumorigenesis of the DFX group was significantly suppressed compared to the control group (Figure 2A). The tumor weight of the DFX group was also significantly suppressed compared to the control group (Figure 2B). Immunohistochemistry and area index analysis revealed that expression of stemness markers (Nanog, Sox2, Klf4, c-Myc) was significantly suppressed compared to the control group (Figure 2C). Body weights of treated mice were not significantly different (Supplementary Figure S1). Thus, DFX suppressed tumorigenesis and expression of stemness markers in tumors derived from miPS cells in vivo.

### 2.3. DFX Suppresses Proliferation and Expression of Stemness Markers in Human Cancer Cell Lines

**Figure 3:**
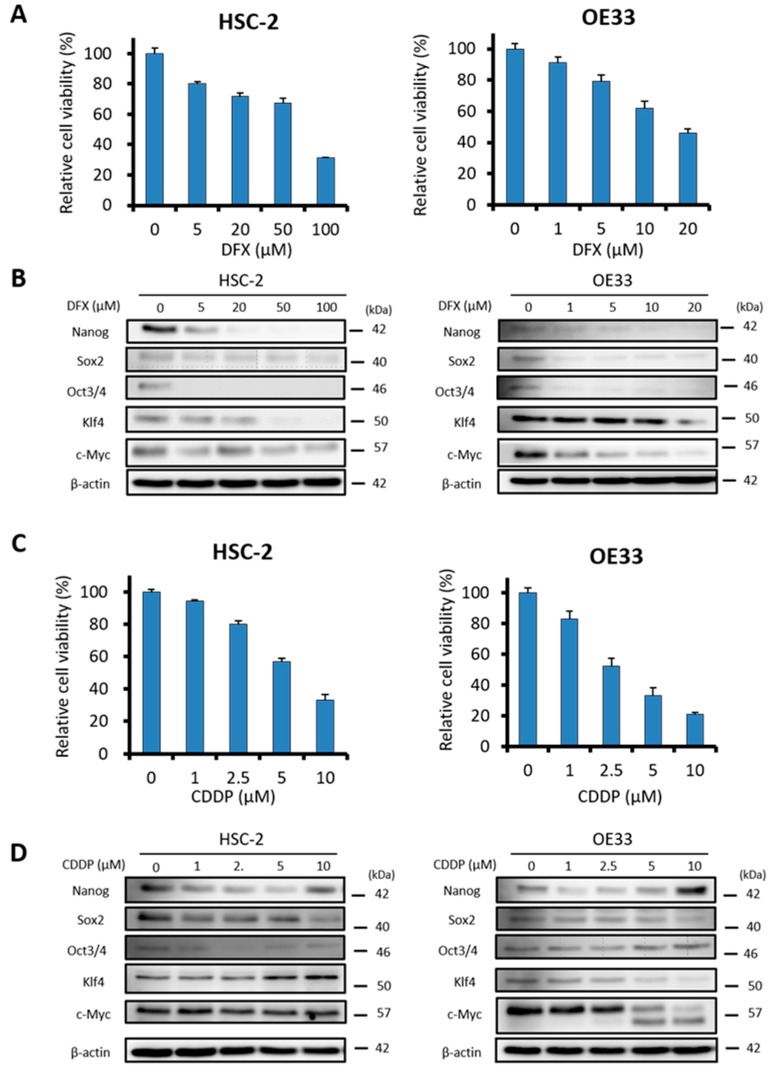
Effect of DFX on proliferation and expression of stemness markers in human cancer cell lines in vitro. (**A**) Cultured HSC-2 cells and OE33 cells were treated with different concentrations of DFX for 48 h, and cell viability was evaluated with the XTT assay. DFX suppressed the proliferation of HSC-2 cells and OE33 cells in a dose-dependent manner. Cell viability in the absence of treatment was set at 100%. (**B**) After culturing HSC-2 cells and OE33 cells with different concentrations of DFX for 48 h, cell lysates were collected, and the total protein was analyzed for expression of the indicated stemness markers with western blot analysis. Expression of stemness markers was suppressed by DFX in a dose-dependent manner. (**C**) Cultured HSC-2 cells and OE33 cells were treated with different concentrations of CDDP for 48 h, and cell viability was evaluated with the XTT assay. CDDP suppressed the proliferation of HSC-2 cells and OE33 cells in a dose-dependent manner. Cell viability in the absence of treatment was set at 100%. (**D**) After culturing HSC-2 cells and OE33 cells with different concentrations of CDDP for 48 h, cell lysates were collected, and the total protein was analyzed for expression of the indicated stemness markers with western blot analysis. Most stemness markers were upregulated or unchanged after treatment with CDDP.

Next, to assess the effect of DFX and CDDP on cytotoxicity and expression of stemness markers in human cancer cell lines, we used HSC-2 cells and OE33 cells, which express similar stemness markers (Nanog, Sox2, Oct3/4, Klf4, c-Myc) as ES cells. The XTT assay showed that DFX suppressed proliferation and expression of stemness markers (Figure 3A,B) in HSC-2 cells and OE33 cells in a dose-dependent manner. CDDP suppressed the proliferation of HSC-2 cells and OE33 cells in a dose-dependent manner (Figure 3C), but expression of some stemness markers remained unchanged or increased (Figure 3D). These results indicated that DFX effectively suppressed both proliferation and stemness in cancer cell lines with high stemness status.

### 2.4. DFX Suppresses Spherogenicity in Human Cancer Cell Lines

**Figure 4:**
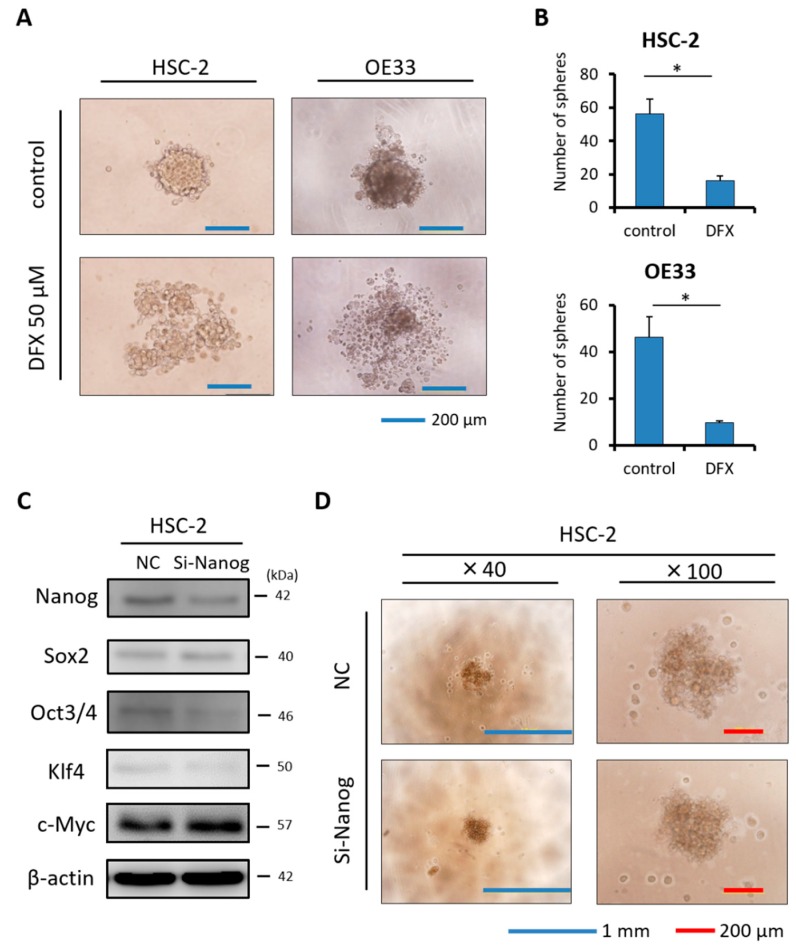
Effect of DFX on spherogenicity of human cancer cell lines and treatment with Nanog siRNA in vitro. (**A**) After treatment with 0.2% DMSO or 50 μM DFX, a single suspension of HSC-2 cells or OE33 cells was used for the sphere formation assay in a 96-well ultra-low attachment plate. DFX suppressed the spherogenicity of HSC-2 cells and OE33 cells. (**B**) A single suspension of HSC-2 cells or OE33 cells as described above was used for the spheroid colony assay in a 24-well ultra-low attachment plate. The number of spheres over 50 μm in diameter was counted. The experiments were performed in triplicate, and means ± S.E.M. of each group are shown. DFX significantly suppressed the number of spheres. * *p* < 0.05. (**C**) HSC-2 cells were transfected with control or si-Nanog for 48 h, and the expression of stemness markers (Nanog, Sox2, Oct3/4, Klf4, c-Myc) was determined with western blot analysis. β-actin was used as a loading control. siRNA suppressed the expression of Nanog, Oct3/4, and Klf4. (**D**) HSC-2 cells were transfected with control or si-Nanog for 48 h, and the sphere formation assay was performed. No differences were found in spherogenicity between the control and si-Nanog cultures.

To explore the effect of DFX on self-renewal, a sphere formation assay was performed. DFX suppressed the spherogenicity of HSC-2 cells and OE33 cells compared to the control group (Figure 4A). Furthermore, the average numbers of tumor spheres derived from HSC-2 cells and OE33 cells treated with DFX were significantly decreased compared to those in the control group (Figure 4B). To investigate the effect of Nanog, which is an upstream factor of some stemness markers [18], on spherogenicity, HSC-2 cells were transfected with small interfering RNA against Nanog (si-Nanog), and its interfering efficiency was measured with western blot analysis. 

Expression of Oct3/4 and Klf4 in addition to Nanog was suppressed by si-Nanog (Figure 4C). However, we observed no difference in spherogenicity of HSC-2 cells after transfection with si-Nanog (Figure 4D). Taken together, DFX suppressed not only Nanog expression but also expression of some other stemness markers such as Sox2, Oct3/4, Klf4, and c-Myc and strongly suppressed stemness, including spherogenicity, of HSC-2 cells and OE33 cells.

### 2.5. Combination Therapy with DFX and Chemotherapy Induces Synergistic Antitumor Effects in Human Cancer Cell Lines and Suppresses Expression of Stemness Markers and Function

**Figure 5:**
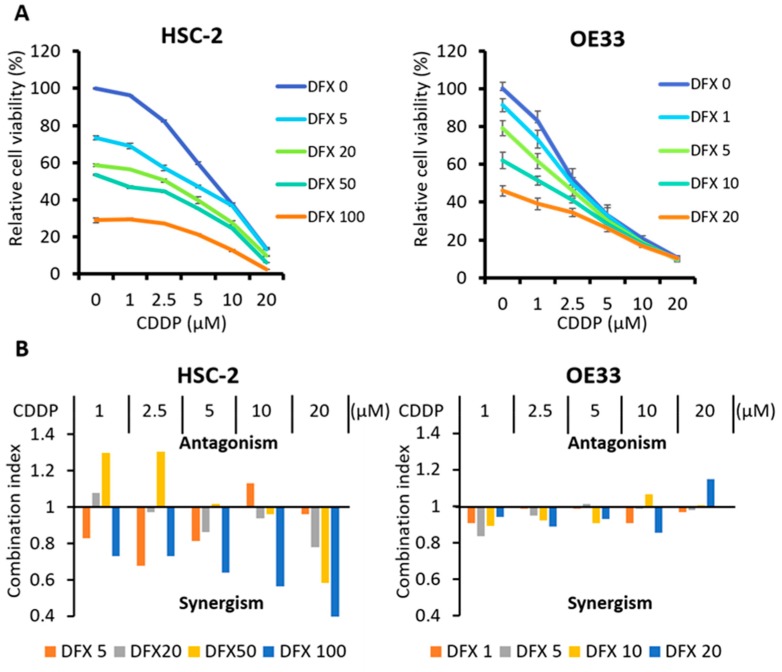

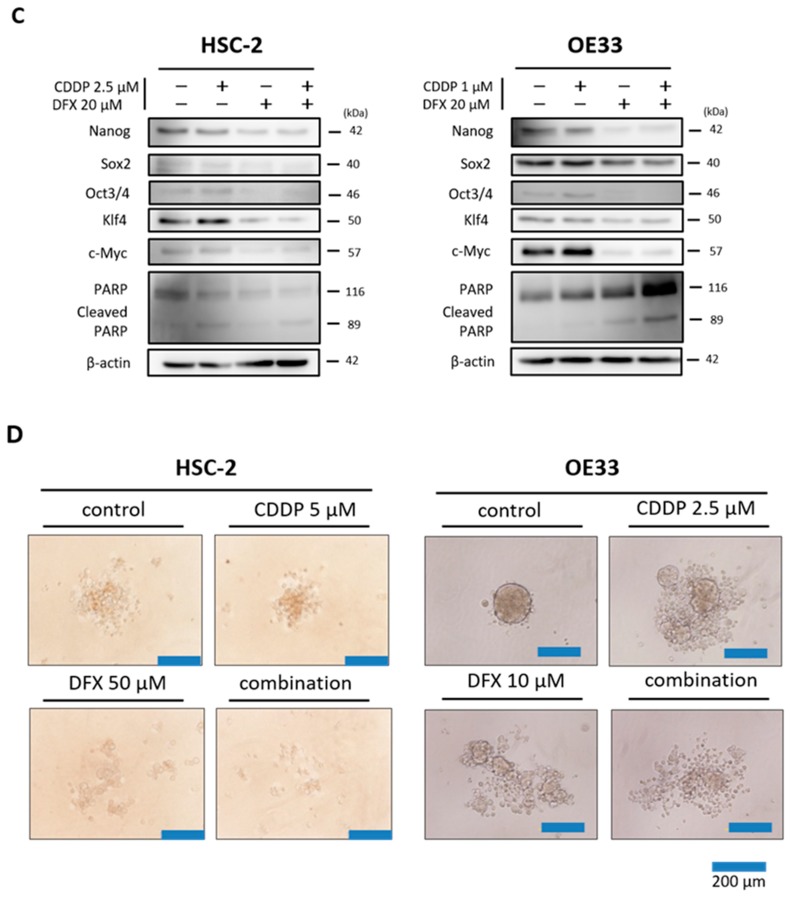
Effect of CDDP and DFX on cell growth and expression of stemness markers and function in vitro. (**A**) Inhibition of cell growth was evaluated using the XTT assay. Combined treatment with CDDP and DFX inhibited the growth of HSC-2 and OE33 cells in a dose-dependent manner compared with single agent treatment. (**B**) The combination index was analyzed with Calcusyn software using the results of the XTT assay. Several drug dose combinations of CDDP and DFX indicated synergism (Combination index < 1.0) of the combination treatment. (**C**) Expression of stemness markers was evaluated with western blot analysis. β-actin was used as a loading control. DFX and combination treatment suppressed the expression of stemness markers (Nanog, Sox2, Oct3/4, Klf4, c-Myc) in HSC-2 and OE33 cells. (**D**) Spherogenecity was evaluated with sphere formation assay in a 96-well ultra-low attachment plate. DFX and combination treatment suppressed the spherogenecity in HSC-2 and OE33 cells.

To evaluate the effect of combination therapy using DFX and CDDP on cytotoxicity, synergy, and stemness in HSC-2 cells and OE33 cells, the XTT assay, combination index, and western blot analysis were performed. The XTT assay showed that cytotoxicity of combination therapy against HSC-2 cells and OE33 cells increased in a dose-dependent manner (Figure 5A). Combination index analysis showed that several drug dose combinations had a synergistic effect against HSC-2 cells and OE33 cells (Figure 5B). Western blot analysis showed that combination therapy suppressed expression of stemness markers and induced apoptosis in HSC-2 cells and OE33 cells to a similar extent as DFX (Figure 5C). Spherogenecity was also suppressed in combination therapy (Figure 5D). These results indicate that combination therapy using DFX and CDDP has stronger cytotoxicity and suppression of stemness markers and function in human cancer cell lines with high stemness status.

### 2.6. Combination Therapy with DFX and Chemotherapy Suppresses Tumor Growth of Human Oral Squamous Carcinoma In Vivo

**Figure 6:**
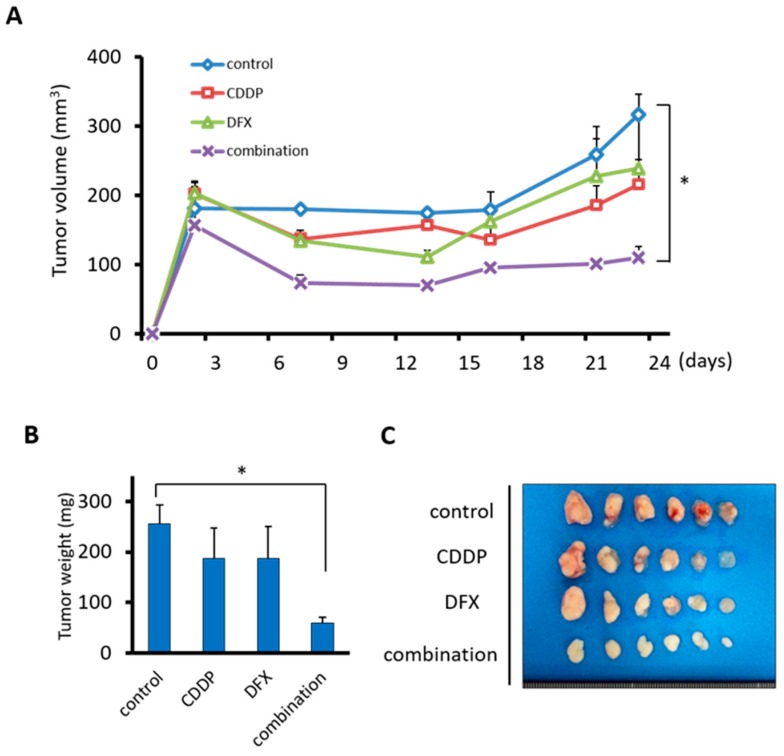
Combination therapy with DFX and CDDP is most effective in suppressing the tumor growth of HSC-2 cells in vivo. (**A**) HSC-2 cells (3 × 10^6^ per mouse) were injected subcutaneously into the right flank of 24 mice. On day 2 when the tumors reached 150~200 mm^3^, mice were randomly assigned to one of four groups (*n* = 6 per group), and the treatments were initiated as indicated. Tumor size was monitored twice per week. The mean tumor volumes of each group ± S.E.M. and *p* values for comparison between groups are shown. In the combination group, tumor growth of HSC-2 cells was most effectively and significantly suppressed compared with the control group. * *p <* 0.05. (**B**) Only the combination therapy significantly suppressed the tumor weight of HSC-2 cells in vivo. * *p* < 0.05. (**C**) All isolated tumors are shown.

To address the effect of combination therapy on tumorigenicity and expression of stemness markers in vivo, we employed the subcutaneous xenograft model of HSC-2 cells by using BALB/c nude mice. DFX was administered orally concerning clinical use. Tumor volumes of control group mice increased during the experimental period. Only the tumor growth of the combination group was significantly decreased compared to the control group (Figure 6A,C). In addition, the tumor weight of the combination group was significantly decreased compared to the control group (Figure 6B). Body weights of treated mice were not significantly different (Supplementary Figure S2). All mice did not reveal significant side effects including bloody urine and rough skin. Thus, combination therapy inhibited growth of tumors derived from HSC-2 cells in vivo.

## 3. Discussion

In our study, we employed miPS cells as a model of ES cells, which possess high stemness status. We also employed HSC-2 cells and OE33 cells as models of heterogeneous cancer tissue that includes CSCs. We focused on the point that some CSCs express similar stemness transcription factors (Nanog, Sox2, Oct3/4, Klf4, c-Myc) as ES cells. These transcription factors are important for the maintenance of pluripotency [19,20,21]. DFX suppressed expression of stemness markers and spherogenesis of miPS cells and human cancer cells and also suppressed tumorigenesis of miPS cells. To test the effect of DFX to suppress stemness for cancer therapy, we also verified the effect of combination therapy using DFX and chemotherapy. In vitro, we confirmed the synergistic effect of combination therapy on cytotoxicity, suppression of expression of stemness markers, and suppression of spherogenesis. In vivo, combination therapy showed a strong antitumor effect. Moreover, toxicity of DFX against human fibroblast cells (WI38, FEF3), which are non-cancerous cells, was minimal, suggesting the potential usefulness of this combination therapy (Supplementary Figure S3).

One problem in current cancer treatment is the existence of CSCs, which are resistant to conventional chemotherapy and radiation and are considered to be related to metastasis and recurrence [22,23]. Some reports have shown that chemotherapy or radiation therapy induces the generation of CSCs [24,25,26,27,28]. CSC targeting therapies have been extensively investigated [22,29], but they are not yet in clinical use. Our results showed that DFX suppressed the stemness in cancer cells with high stemness status and that combination therapy with chemotherapy may be a novel approach against CSCs. Our group has reported that DFX suppresses stemness and tumorigenesis in a CSC model [30]. In our current study, we have shown that this phenomenon is general and can be applied in clinical use. We verified the effect of combination therapy with DFX and CDDP and showed that DFX suppressed the stemness of cancer cells with high stemness status in heterogeneous cancer tissue and that damage may be specifically directed toward these cancer cells with high stemness. Furthermore, apoptosis was enhanced by adding CDDP to DFX. The mechanism by which DFX suppresses stemness is unclear, but we suggest that iron metabolism may be involved in the pathway of expression of stemness genes. According to previous reports, expression of Nanog is related to the Stat3 signaling pathway [31,32,33]. Our result in which DFX suppressed the expression of Stat3 (Supplementary Figure S4) suggests that DFX may regulate the expression of Nanog.

Raggi et al. reported that iron metabolism is related to stemness of cholangiocarcinoma stem-like cells [34]. They showed that cholangiocarcinoma stem-like cells express high levels of ferritin and low levels of transferrin receptor 1 and ferroportin. We evaluated these iron-related markers after DFX treatment and observed that DFX downregulated the expression of ferroportin and ferritin and upregulated the expression of transferrin receptor (Supplementary Figure S5A,B). We also evaluated the effect of DFX on CD44 positive cell ratio using flow cytometry. DFX decreased the CD44 positive cell ratio (Supplementary Figure S6A). The mRNA of Nanog was suppressed by DFX in a dose-dependent manner (Supplementary Figure S6B). These result suggests that DFX suppressed the population of stem-like cells in HSC-2 cells and OE33 cells. Our observation that si-Nanog did not suppress the spherogenicity of HSC-2 cells suggests that knockdown of Nanog is insufficient to suppress stemness. On the contrary, the result in which DFX suppressed almost all stemness markers we examined indicates that DFX may interrupt the stemness network.

As a therapeutic strategy for cancer and CSCs, attention is recently focused on iron metabolism [35,36,37]. We confirmed some basic effects by iron chelator. DFX suppressed the migration ability of CSC (Supplementary Figure S7). The ability to induce the secretion of vascular endothelial growth factor (VEGF) in cancer cells was also revealed (Supplementary Figure S8). These results which indicated that CSCs had a tendency to escape from an iron-depleted condition are in line with our previous reports [7,38].

Our study has the following limitations. Although we confirmed that DFX suppressed the expression of stemness markers in CSCs, DFX did not recognized the CSCs from cell surface antigen. CD44 antibody includes both standard and variant isoforms. We did not check the ratio of CD44 variant isoform. We employed bulk cells as a model of heterogeneous cancer tissue and only focused on the effect of DFX against CSCs. In addition, we did not evaluate the relapse in vivo. Further studies are needed to evaluate the effect of DFX on other stemness markers.

We also need to confirm the status of cells after treatment with DFX. DFX may induce specific cytotoxicity against cells with high stemness status or may lead to differentiation of such cells. Either way, the fact that DFX suppressed stemness in cancer tissue is important and suggests that DFX may be a useful treatment option. Thus, clarification of the mechanism is urgently needed.

In conclusion, regulating iron metabolism may be a novel strategy via suppressing stemness for CSC targeted therapy.

## 4. Materials and Methods

### 4.1. Cell Lines and Cell Culture

miPS cells were purchased from Riken Cell Bank (RIKEN BRC, Ibaraki, Japan). The human oral squamous carcinoma cell line (HSC-2) was obtained from Showa University. The human esophageal adenocarcinoma cell line (OE33) was purchased from The European Collection of Authenticated Cell Cultures (ECACC, Salisbury, UK). We also used the human fibroblast cell lines, FEF3 and WI38, as representative cells with a “normal” non-cancerous phenotype. FEF3 cells were isolated from human fetal esophagus as described previously [39]. WI38 fetal lung human fibroblasts were purchased from the Health Science Research Resource Bank (Osaka, Japan). All cells were incubated at 37 °C in a humidified atmosphere containing 5% CO_2_.

miPS cells were maintained in medium (Dulbecco’s modified eagle medium (DMEM) containing 15% fetal calf serum (FCS), 0.1 mM non-essential amino acids, 2 mM l-glutamine, 0.1 mM 2-mercaptoethanol, 1000 U/mL leukemia inhibitory factor, 50 U/mL penicillin, and 50 U/mL streptomycin) on feeder layers of mitomycin C-treated mouse embryonic fibroblast cells (Reprocell, Kanagawa, Japan). HSC-2, FEF3, and WI38 cells were maintained in DMEM containing 10% FCS, 50 U/mL penicillin, and 50 U/mL streptomycin. OE33 cells were maintained in RPMI containing 10% FCS, 50 U/mL penicillin, and 50 U/mL streptomycin.

### 4.2. Reagents

Deferasirox (DFX, EXJADE) was obtained from Novartis Pharma (Tokyo, Japan). For in vitro studies, DFX was dissolved in DMSO (Sigma-Aldrich, St. Louis, MO, USA) at a stock concentration of 50 mM. For in vivo studies, DFX was dissolved in saline. Cisplatin (CDDP, Randa) was purchased from Nippon Kayaku (Tokyo, Japan) and dissolved in phosphate-buffered saline (PBS).

### 4.3. Cell Viability Assay

The XTT assay (Cell Proliferation kit II, Roche, Mannheim, Germany) was used to assess cell proliferation according to the manufacturer’s protocol. The cells were seeded in 96-well plates and treated with DFX and/or CDDP for 48 h at 37 °C. The combination index was calculated with CalcuSyn software (BioSoft, Inc., Cambridge, UK). We seeded the cells as follows: HSC-2 (6.0 × 10^3^/well), OE33 (3.0 × 10^3^/well), FEF3 and WI38 (2.0 × 10^3^/well).

### 4.4. Sphere Formation Assay

Cells were seeded in 96-well ultra-low attachment plates (Corning Costar, Sigma-Aldrich) at a density of 5 × 10^2^ cells/well and maintained in DMEM/nutrient mixture F-12 (Sigma) containing B-27 supplement (Invitrogen, Carlsbad, CA, USA), 20 ng/mL epidermal growth factor (Sigma), 10 ng/mL fibroblast growth factor (Sigma), 0.4% bovine serum albumin (Sigma), and 5 μg/mL insulin (Invitrogen) for 7 days.

### 4.5. Live/Dead Assay

The Live/Dead viability assay was performed to assess cell viability of miPS cells after treatment with DFX. The miPS cells were treated with 0.2% DMSO or 50 μM DFX for 48 h, rinsed with PBS, and incubated with Calcein AM (Thermo Fisher Scientific, Waltham, MA, USA) and Ethidium homodimer-1 (Thermo Fisher Scientific) according to the manufacturer’s protocol. Cells were visualized using a fluorescence microscope (Olympus IX71, Olympus, Tokyo, Japan).

### 4.6. Nanog Small Interfering RNA Transfection

To confirm the effect of Nanog on spherogenicity, HSC-2 cells were transfected with Silencer select siRNA against NANOG (catalog no. s36650; Ambion, Life Technologies, Carlsbad, CA, USA) or scrambled control (Silencer Negative Control, Ambion, Life Technologies) using Lipofectamine RNAiMAX (Invitrogen) at a final concentration of 20 nM and 10 nM, respectively. Sphere growth was initiated 48 h post-transfection.

### 4.7. Western blotting

Protein was extracted from whole cells after 48 h of incubation in medium and reagents. The concentrations of extracted protein were measured using standard protocols. Cells were lysed using cell lysis buffer (50 mmol/L Tris-HCl (pH 7.4), 30 mmol/L NaCl, and 1% Triton X-100) containing protease inhibitors (cOmplete Mini, Roche Diagnostics GmbH, Basel, Switzerland). Equal amounts of total cellular proteins (50 μg/lane) were separated by sodium dodecyl sulfate-polyacrylamide gel electrophoresis and transferred electrophoretically to polyvinylidene difluoride filter membranes (GE Healthcare UK Ltd., Buckinghamshire, UK) according to the manufacturer’s protocol. The following primary antibodies were used: anti-Nanog antibody (catalog no. 4903S; Cell Signaling Technology, Danvers, MA, USA), anti-Sox2 antibody (catalog no. ab97959; Abcam, Cambridge, MA, USA), anti-Oct3/4 antibody (catalog no. MAB1759; R&D Systems, Minneapolis, MN, USA), anti-KLF4 antibody (catalog no. ab72543; Abcam), anti-c-Myc antibody (catalog no. ab32072; Abcam), anti-transferrin receptor antibody (catalog no. ab84036; Abcam), anti-DMT1 antibody (catalog no. ab123085; Abcam), anti-ferroportin/SLC40A1 antibody (catalog no. NBP1-21502; Novus Biologicals, Littleton, CO, USA), anti-ferritin heavy chain antibody (catalog no. ab65080; Abcam), anti-β-actin antibody (catalog no. A5441; Sigma-Aldrich), anti-PARP antibody (catalog no. 9542; Cell Signaling Technology), anti-cleaved caspase-3 antibody (catalog no. 9664; Cell Signaling Technology), anti-caspase 3 antibody (catalog no. sc-7148; Santa Cruz, Dallas, TX, USA), anti-Stat3 antibody (catalog no. 12640; Cell Signaling Technology), and anti-phospho-Stat3 antibody (catalog no. 9145; Cell Signaling Technology). All primary antibodies were used at a 1:1000 dilution. The following secondary antibodies were used: anti-Mouse IgG, HRP-Linked Whole antibody Sheep (catalog no. NA931; GE Healthcare, UK Ltd.), anti-Rabbit IgG, HRP-Linked Whole antibody Donkey (catalog no. NA934; GE Healthcare, UK Ltd.), anti-Rat IgG, HRP-Linked Whole antibody Goat (catalog no. NA935; GE Healthcare, UK Ltd.). All secondary antibodies (GE Life Sciences) were used at a 1:2500 dilution. The membranes were incubated with primary antibodies overnight at 4 °C, followed by incubation with secondary antibodies. ECL prime Western Blotting Detection Reagent (GE Healthcare UK Ltd.) was used to detect the peroxidase activity of secondary antibodies. Membranes were probed for β-actin as a loading control, and all sample data values were normalized to the corresponding control data values. Densitometric analysis was performed using Image J software (NIH).

### 4.8. Flow Cytometry Analysis

HSC-2 and OE-33 cells were seeded at 5 × 10^4^ cells/mL in 6 well plates 24 h before treatment with different concentrations of DFX for 48h, after which cells and medium were recovered, centrifuged (5 min, 400 g, 4 °C). Cells were suspended in PBS containing 2% fetal bovine serum and 0.1% sodium azide, and stained with the anti-mouse/human CD44 Antibody (Cat. No.103015, BioLegend, San Diego, CA, USA) and propidium iodide staining (Life Technologies Corporation) after 10 min of pre-incubation with human TruStain FcX (Fc Receptor Blocking Solusion, BioLegend Cat. No. 422301). Cell fluorescence was detected with MACSQuant Analyzer (Miltenyi Biotec, Bergisch Gladbach, Germany) and analyzed with MACSQuantify Software.

### 4.9. Real-Time Quantitative PCR

HSC-2 and OE-33 cells were seeded at 5 × 10^4^ cells/mL in 6 well plates 24 h before treatment with different concentrations of DFX for 48 h. Total RNA was isolated from HSC-2 and OE-33 cells using Trizol Reagent (Gibco BRL, Grand Island, NY, USA) and High Pure RNA Isolation kit (Roche Applied Science, Basel, Switzerland), respectively. First-strand cDNA was constructed from total RNA using the oligo (dT) primer. Real-time quantitative PCR analysis was performed using StepOne with Taqman PCR master mix (Applied Biosystems, Foster City, CA, USA). The primers used in this study were: GAPDH (Applied Biosystems) and Nanog (Integrated DNA Technologies, Coralville, USA). The quantification of the gene of interest was normalized to GAPDH and expressed as fold-increases relative to the negative control for each treatment at each time point as previously described.

### 4.10. VEGF ELISA Assay

To evaluate the supernatant VEGF secreted by HSC-2 and OE33 cells, we used a VEGF enzyme-linked immunosorbent assay (ELISA) kit (Proteintech, Rosemont, IL, USA). The cancer cells were plated in 6 well plates and were treated with different concentrations of DFX. After a 48-h treatment, the supernatant and cells were harvested and VEGF content was assayed by ELISA according to the protocol provided by the manufacturer.

### 4.11. Tumor Xenograft Model and Experiment

All animal experiments were performed according to the Japanese Welfare and Management of Animals Act and conducted in accordance with institutional guidelines at Shigei Medical Research Institute, Okayama, Japan. All animal experiments were approved by the Ethics Review Committee for Animal Experimentation of Shigei Medical Research Institute (#160401-1), Okayama, Japan. Female BALB/c (nu/nu) mice were purchased from CLEA Japan (Tokyo, Japan). Female BALB/c (nu/nu) mice were purchased from CLEA Japan. The experiment started when the mice were 9 weeks of age. HSC-2 cells in culture were harvested and resuspended in a 1:1 ratio of PBS and Matrigel (BD Biosciences). HSC-2 cells (3.0 × 10^6^) were injected subcutaneously into the right flank. When the tumor size reached 150~200 mm^3^, the mice were randomly divided into four groups (control group, CDDP group, DFX group, combination group, *n* = 6 per group). Each group was treated with oral gavage of saline or DFX (160 mg/kg) three times per week for 3 weeks and by intraperitoneal injection of saline or CDDP (6 mg/kg) once per week for 3 weeks. Tumor size and body weight were measured every 3 days. The tumor volume (mm^3^) was calculated with the formula d2 × D/2 where d and D are the shortest and longest diameters in mm, respectively. At the end of the experiment, the mice were sacrificed, and the tumors were excised, weighed, and processed for histological analysis.

### 4.12. Immunohistochemistry of In Vivo-Derived Tumor Tissues

Harvested tumors were fixed in 10% paraformaldehyde and embedded in paraffin prior to immunostaining. The same anti-Nanog antibody, anti-Sox2 antibody, anti-Oct3/4 antibody, anti-KLF4 antibody, and anti-c-Myc antibody were used as described in the western blot analysis section. Evaluation of the Nanog, Sox2, Oct3/4, KLF4, and c-Myc area index was performed with Image J software (http://rsb.info.nih.gov/ij/).

### 4.13. Statistical Analysis

All statistical analyses were performed with SPSS advanced statistics 16.0 software (SPSS, Tokyo, Japan). For two-group comparisons, Student’s *t*-test was used. For multiple-group comparisons, analysis of variance with Tukey’s test was used. *p* values < 0.05 were considered statistically significant.

## 5. Conclusions

In conclusion, to the best of our knowledge, iron metabolism appears important for maintenance of stemness in cell lines with high stemness status including CSCs. The expression of stemness markers such as *Nanog*, *Oct3/4*, *Sox2*, *Klf4*, and *c-Myc* was suppressed by iron chelator. By using iron chelator, regulating iron metabolism and combined with chemotherapy may be a novel strategy for CSC targeted therapy via suppressing stemness.

## Supplementary Materials

The following are available online at http://www.mdpi.com/2072-6694/11/2/177/s1, Figure S1: miPS cells (5 × 105 per mouse) treated with 0.2% DMSO or 50 μM DFX were implanted subcutaneously into the right flank, and tumorigenicity was evaluated. There were no significant differences between groups. Data are represented as average ± S.E.M. (*n* = 5), Figure S2: HSC-2 cells (3 × 106 per mouse) were injected subcutaneously into the right flank of 24 mice. On day 2 when the tumors reached 150~200 mm^3^, mice were randomly assigned to one of four groups (*n* = 6 per group), and the treatments were initiated as indicated. The mean body weight of each group ± S.E.M. for comparison between groups are shown. There were no significant differences between groups, Figure S3: Cultured WI38 cells and FEF3 cells were treated with different concentrations of DFX for 48 h, and cell viability was evaluated with the XTT assay. The cytotoxity of DFX against WI38 cells and FEF3 was very low. Cell viability in the absence of treatment was set at 100%, Figure S4: Experiment of Nanog, Stat3 and phosho-Stat3 was evaluated with western blot analysis. β-actin was used as a loading control. DFX and combination treatment suppressed the expression of Nanog, Stat3 and phosho-Stat3 in HSC-2 cells, Figure S5: (A) Experiment of iron-related markers (FPN, FtH, TfR, DMT-1) was evaluated with western blot analysis. β-actin was used as a loading control. DFX suppressed the expression of iron-related markers except TfR in HSC-2 and OE33 cells. (B) Densitometric analysis of western blot also showed that DFX suppressed the expression of iron-related markers except TfR in HSC-2 and OE33 cells. Statistical significance was determined as * *p* ≤ 0.05, Figure S6: (A) HSC-2 and OE33 cells treated with different concentrations of DFX for 48 h were analyzed by flow cytometry. CD44 antibody was used as a stemness marker. Living CD44 positive cells ratio was decreased by DFX. (B) Total RNA of HSC-2 and OE33 cells treated with DFX with indicated concentrations were used in the PCR analysis. The mRNA of Nanog was suppressed by DFX in a dose-dependent manner. Relative expression level in the absence of treatment was set at 1. Statistical significance was determined as * *p* ≤ 0.05, Figure S7: (A) Experiment of migration ability was performed with scratch assay. HSC-2 and OE33 cells were seeded in 6well plates and treated with different concentrations of DFX. Migration ability was evaluated with indicated time. DFX suppressed the migration ability in HSC-2 and OE33 cells. (B) Scratch assay was quantitively analyzed as area of gap with Image J software. * *p* < 0.05. Scratched area was remained by DFX in a dose-dependent manner, Figure S8: Experiment of vascular endothelial growth factor secretion was performed with ELISA assay. The supernatant was collected with indicated concentration of DFX. DFX induced vascular endothelial growth factor secretion in HSC-2 and OE33 cells.
