# Supplementary material for: A Novel Combination Cancer Therapy with Iron Chelator Targeting Cancer Stem Cells via Suppressing Stemness
Source: Cancers (Basel). 2019 Feb 3;11(2):177. doi: 10.3390/cancers11020177 (PMC6406536; doi:10.3390/cancers11020177)
Supplement: Supplementary file 1 [file cancers-11-00177-s001.pdf]

cancers

Supplementary Materials: A Novel Combination Cancer Therapy with Iron Chelator Targeting Cancer Stem Cells via Suppressing Stemness

Yuki Katsura, Toshiaki Ohara, Kazuhiro Noma, Takayuki Ninomiya, Hajime Kashima, Takuya Kato, Hiroaki Sato, Satoshi Komoto, Toru Narusaka, Yasuko Tomono, Boyi Xing, Yuehua Chen, Hiroshi Tazawa, Shunsuke Kagawa [1], Yasuhiro Shirakawa, Tomonari Kasai, Masaharu Seno, Akihiro Matsukawa and Toshiyoshi Fujiwara

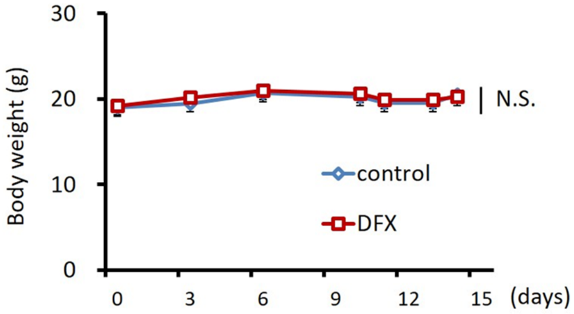

Figure S1. miPS cells (5×10^5 per mouse) treated with 0.2% DMSO or 50 μM DFX were implanted subcutaneously into the right flank, and tumorigenicity was evaluated. There were no significant differences between groups. Data are represented as average ± S.E.M. ($n = 5$).

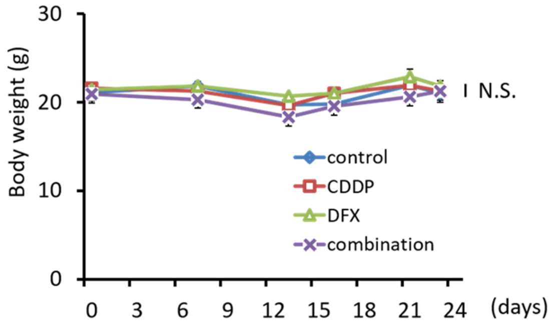

Figure S2. HSC-2 cells (3×10^6 per mouse) were injected subcutaneously into the right flank of 24 mice. On day 2 when the tumors reached 150~200 mm^3, mice were randomly assigned to one of four groups ($n = 6$ per group), and the treatments were initiated as indicated. The mean body weight of each group ± S.E.M. for comparison between groups are shown. There were no significant differences between groups.

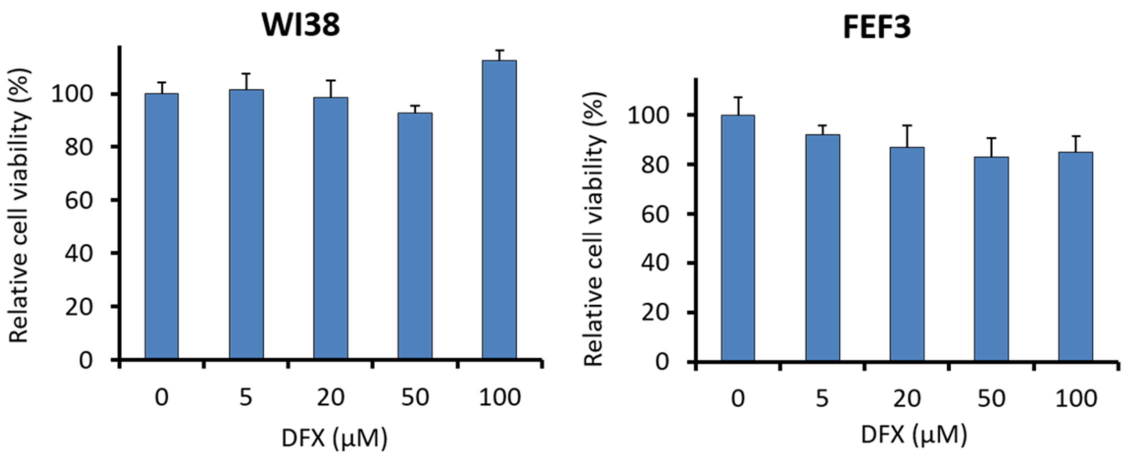

Figure S3. Cultured WI38 cells and FEF3 cells were treated with different concentrations of DFX for 48 h, and cell viability was evaluated with the XTT assay. The cytotoxity of DFX against WI38 cells and FEF3 was very low. Cell viability in the absence of treatment was set at 100%.

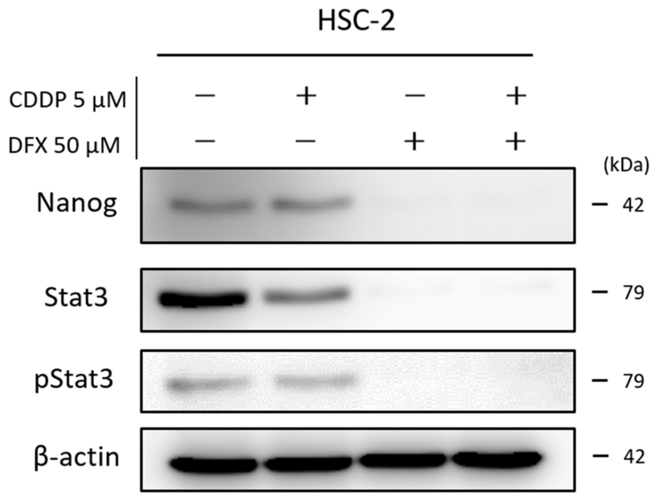

Figure S4. Experiment of Nanog, Stat3 and phosho-Stat3 was evaluated with western blot analysis. β-actin was used as a loading control. DFX and combination treatment suppressed the expression of Nanog, Stat3 and phosho-Stat3 in HSC-2 cells.

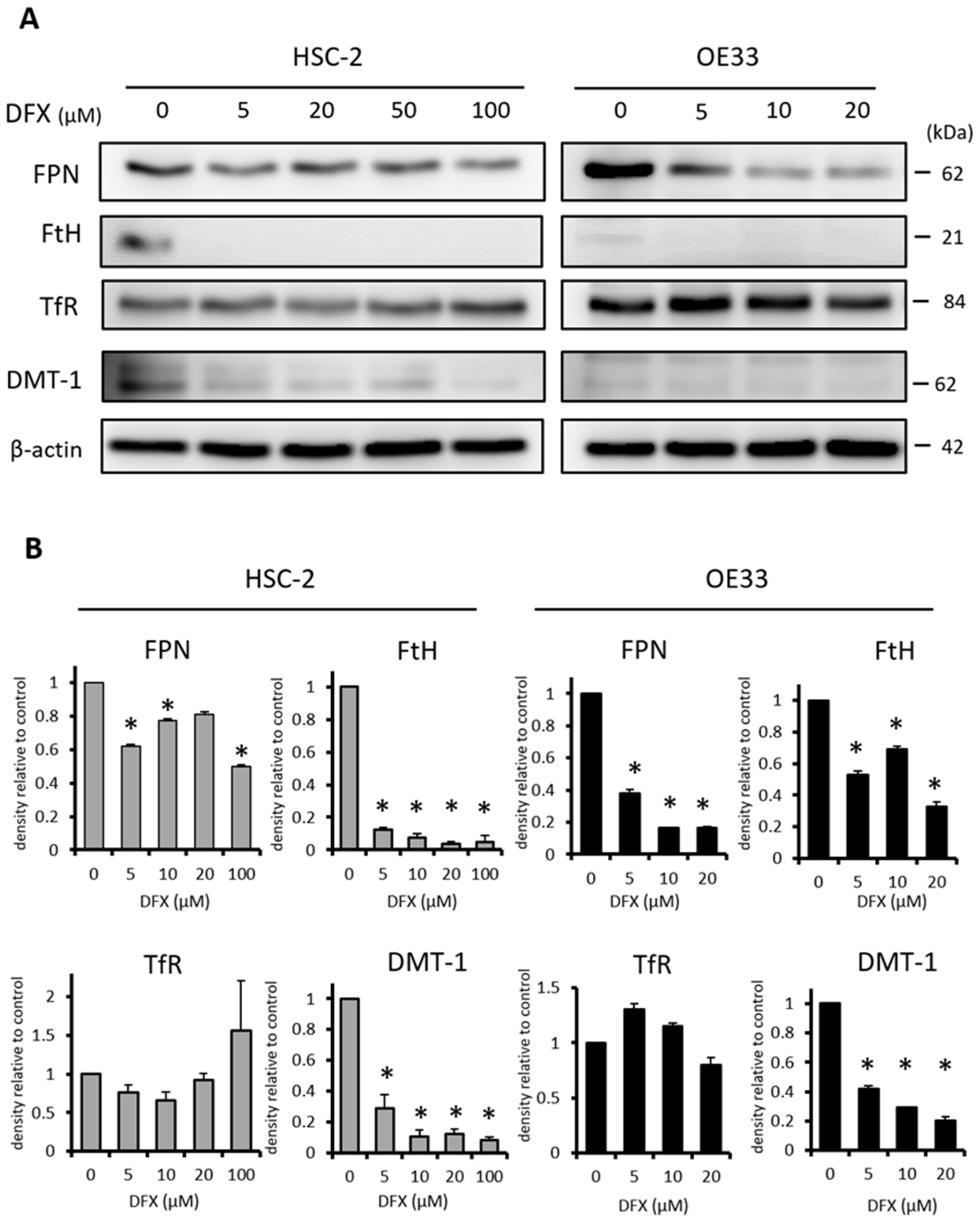

Figure S5. (**A**) Experiment of iron-related markers (FPN, FtH, TfR, DMT-1) was evaluated with western blot analysis. β-actin was used as a loading control. DFX suppressed the expression of iron-related markers except TfR in HSC-2 and OE33 cells. (**B**) Densitometric analysis of western blot also showed that DFX suppressed the expression of iron-related markers except TfR in HSC-2 and OE33 cells. Statistical significance was determined as * $p \leq 0.05$.

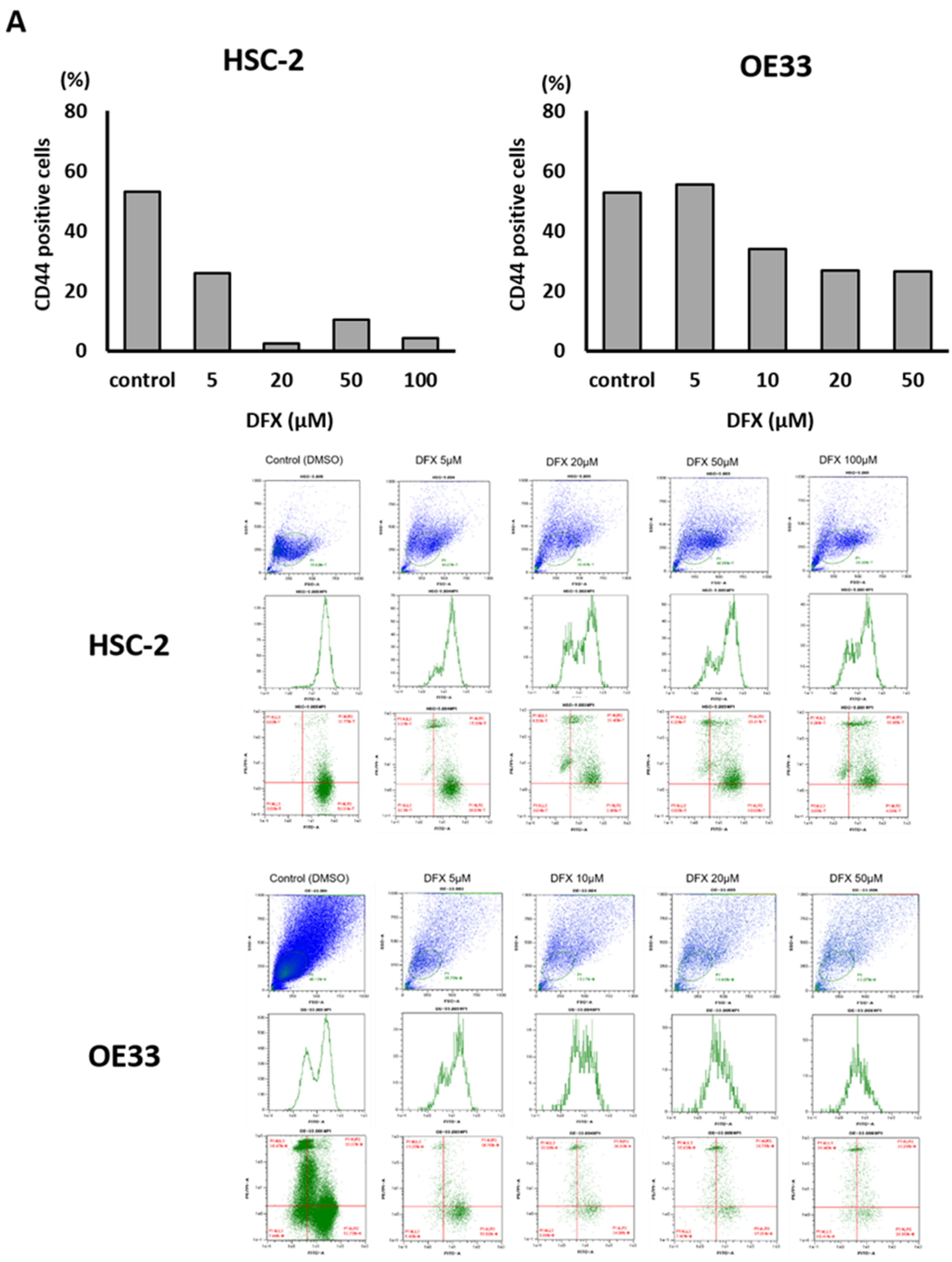

Figure S6. *Cont.*

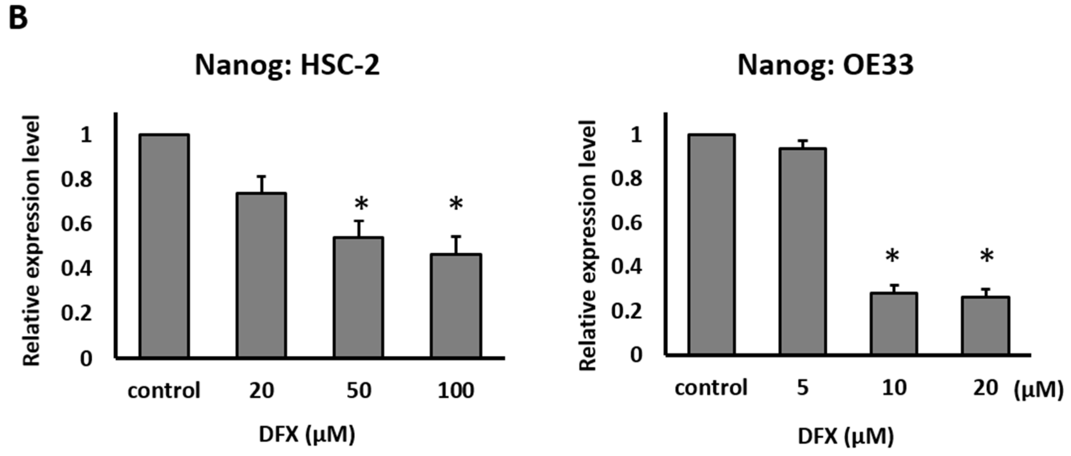

Figure S6. (**A**) HSC-2 and OE33 cells treated with different concentrations of DFX for 48 h were analyzed by flow cytometry. CD44 antibody was used as a stemness marker. Living CD44 positive cells ratio was decreased by DFX. (**B**) Total RNA of HSC-2 and OE33 cells treated with DFX with indicated concentrations were used in the PCR analysis. The mRNA of Nanog was suppressed by DFX in a dose-dependent manner. Relative expression level in the absence of treatment was set at 1. Statistical significance was determined as * $p \leq 0.05$.

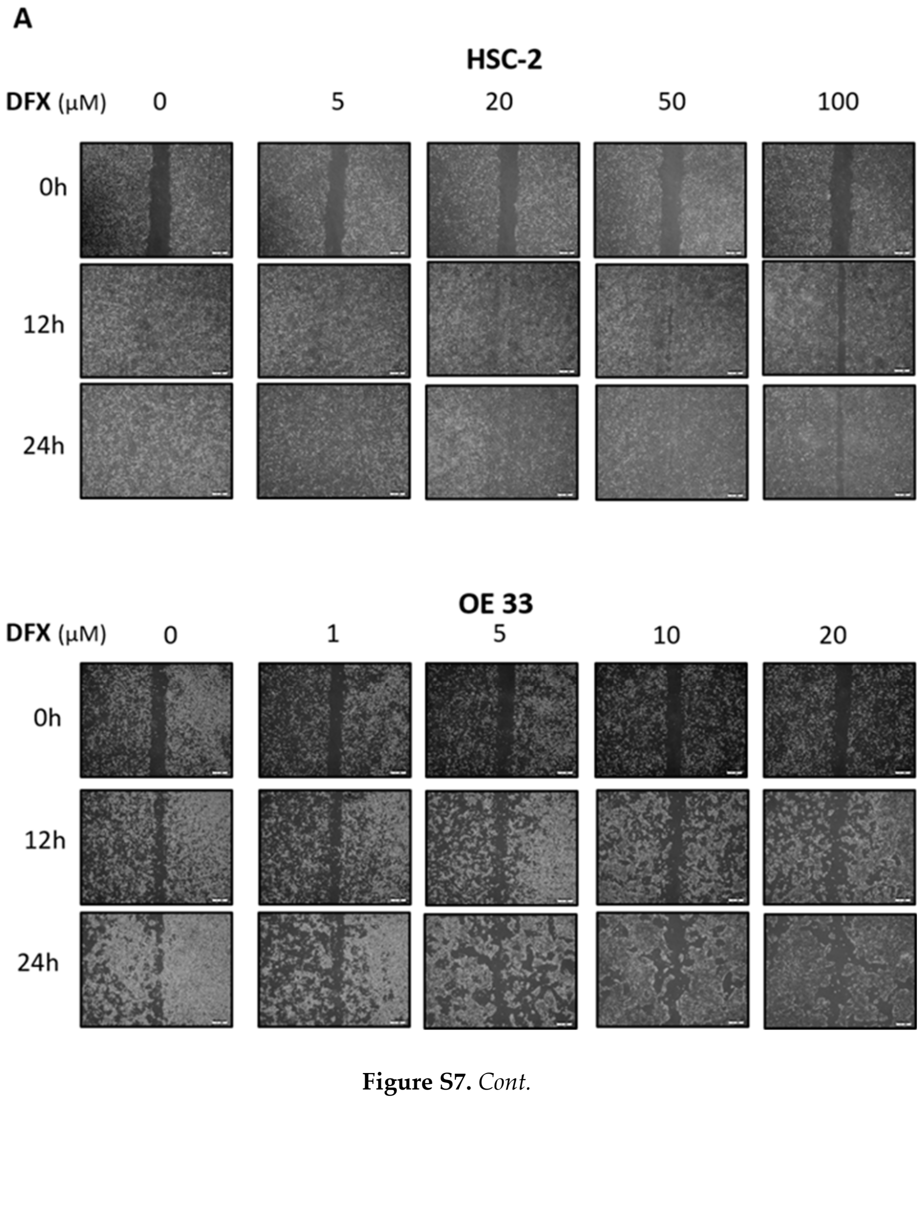

Figure S7. *Cont.*

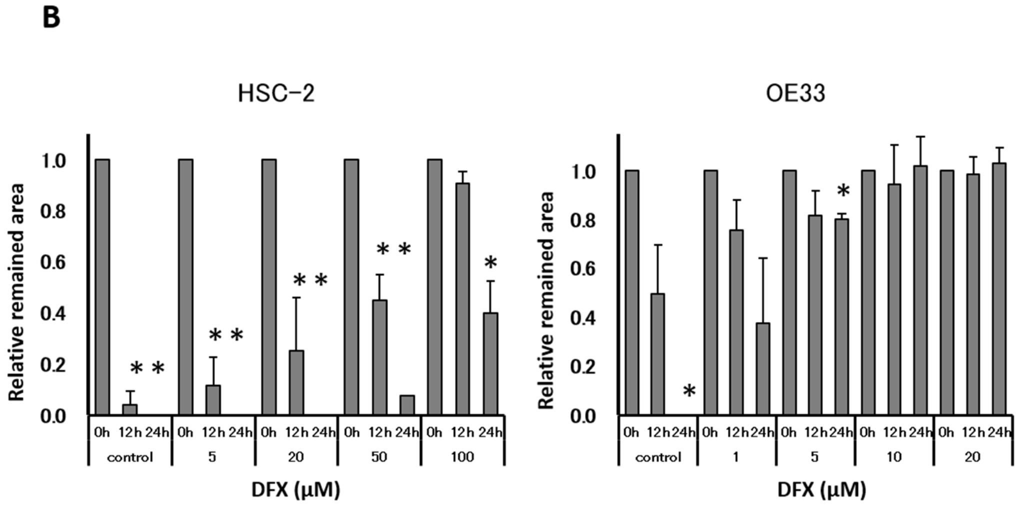

Figure S7. (**A**) Experiment of migration ability was performed with scratch assay. HSC-2 and OE33 cells were seeded in 6well plates and treated with different concentrations of DFX. Migration ability was evaluated with indicated time. DFX suppressed the migration ability in HSC-2 and OE33 cells. (**B**) Scratch assay was quantitively analyzed as area of gap with Image J software. * $p < 0.05$. Scratched area was remained by DFX in a dose-dependent manner.

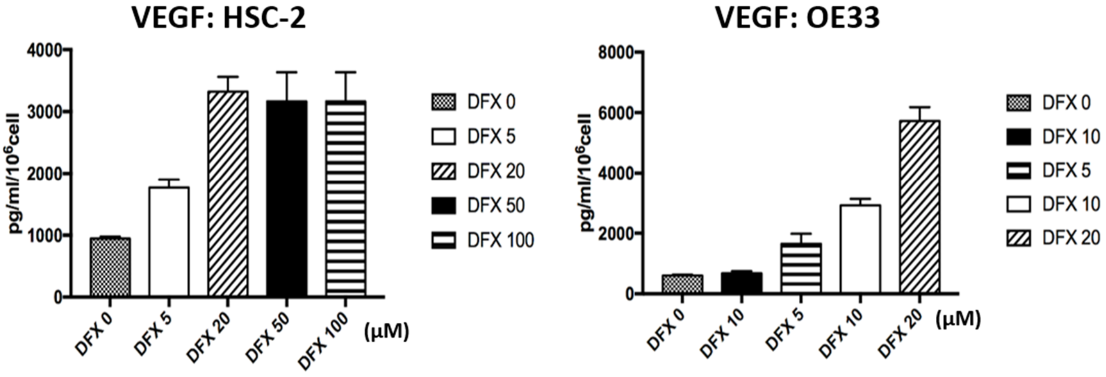

Figure S8. Experiment of vascular endothelial growth factor secretion was performed with ELISA assay. The supernatant was collected with indicated concentration of DFX. DFX induced vascular endothelial growth factor secretion in HSC-2 and OE33 cells.

© 2019 by the authors. Licensee MDPI, Basel, Switzerland. This article is an open access article distributed under the terms and conditions of the Creative Commons Attribution (CC BY) license (http://creativecommons.org/licenses/by/4.0/).
